# Mentorship advances antimicrobial use surveillance systems in low- and middle-income countries

**DOI:** 10.1093/jacamr/dlae212

**Published:** 2024-12-26

**Authors:** Kiran Sunder Bajracharya, Susan Luu, Ron Cheah, Santosh KC, Atifa Mushtaq, Marjorie Elijah, Bhupendra Kumar Poudel, Celeste Fernandes Xavier Cham, Shyamu Mandal, Stephen Muhi, Kirsty Buising

**Affiliations:** Department of Drug Administration, Ministry of Health and Population, Bijulibazar, Kathmandu, Nepal; WHO Collaborating Centre for Antimicrobial Resistance, University of Melbourne at The Peter Doherty Institute for Infection and Immunity, 792 Elizabeth St, Melbourne, VIC 3000, Australia; WHO Collaborating Centre for Antimicrobial Resistance, University of Melbourne at The Peter Doherty Institute for Infection and Immunity, 792 Elizabeth St, Melbourne, VIC 3000, Australia; Department of Drug Administration, Ministry of Health and Population, Bijulibazar, Kathmandu, Nepal; The Drugs Control and traditional Medicine Division, National Institute of Health, Park Rd Chak Shehzad, Islamabad, Pakistan; National Department of Health, Medicine Supplies Procurement and Distribution Branch, PO Box 807, AOPI Building, Waigani Drive, Port Moresby, Papua New Guinea; Pokhara Academy of Health Sciences, Western Regional Hospital, Pokhara, Nepal; Pharmacovigilance Department, National Directorate of Pharmacy and Medicines, Ministry of Health, Lahane, Dili, Timor-Leste; National Academy of Medical Sciences, Bir Hospital, Mahaboudha, Kathmandu, Nepal; WHO Collaborating Centre for Antimicrobial Resistance, University of Melbourne at The Peter Doherty Institute for Infection and Immunity, 792 Elizabeth St, Melbourne, VIC 3000, Australia; WHO Collaborating Centre for Antimicrobial Resistance, University of Melbourne at The Peter Doherty Institute for Infection and Immunity, 792 Elizabeth St, Melbourne, VIC 3000, Australia

## Abstract

A shortage of trained personnel poses significant challenges to implementing antimicrobial use (AMU) surveillance systems in low- and middle-income countries (LMICs). Traditional training models, such as workshops, seminars and online courses, often lack the sustained engagement and support necessary for deep learning and skill mastery. This article advocates for mentorship as an effective training method for AMU professionals in LMICs. Drawing on our experiences as mentors and mentees from 1- to 2-year mentorship programmes in Nepal, Pakistan, Papua New Guinea and Timor-Leste between 2019 and 2023, we highlight the challenges and success factors of mentorship. Our insights demonstrate mentorship’s value in building expertise and sustaining capacity in AMU surveillance, offering a promising solution to address the personnel shortage in these regions.

Surveillance of antimicrobial use (AMU) is critical for implementing evidence-based antimicrobial stewardship (AMS) programmes, guiding treatment decisions and informing public health policies.^1^ This is particularly important in low- and middle-income countries (LMICs), where limited resources, inadequate data infrastructure and a shortage of trained personnel pose significant challenges to implementation efforts.^2–4^

Despite these challenges, recent initiatives such as the WHO’s Global Antimicrobial Resistance and Use Surveillance System (GLASS)^5^ and the Global Point Prevalence Survey^6^ have made progress in AMU surveillance in some LMICs. To sustain this progress and address ongoing challenges, the development of a skilled workforce through targeted training programmes in LMICs is essential.

Current training models in AMU surveillance often do not provide the sustained engagement and support necessary for deep learning and skill mastery, instead relying heavily on short-term methods like workshops, seminars and online courses. Recognizing this problem, mentorship emerges as a potentially more impactful approach. It has proven highly effective in training healthcare professionals^7–9^ and scientists^10,11^ in high-income countries and shows promise for adaptation in LMICs.^12,13^ While several models of mentorship are discussed in the literature, we support a flexible teaching and learning process,^12^ using the Dyad Model,^9^ where an experienced mentor guides the mentee in developing and reassessing their ideas, learning and professional development.^11^

The Fleming Fund Fellowship Scheme^14^ is an exemplar of using mentorship for capacity building in AMU surveillance in LMICs. Between 2019 and 2023, seven health professionals completed 1- to 2-year AMU surveillance fellowships through the Fleming Fund, supported by a team of mentors based at the WHO Collaborating Centre for Antimicrobial Resistance (WHO CC for AMR) at the Peter Doherty Institute for Infection and Immunity in Melbourne, Australia. Four fellows were from Nepal and one each from Pakistan, Papua New Guinea and Timor-Leste. They were selected based on relevant work experience and their ability to apply acquired knowledge and skills directly to their current management, policymaking or research roles within their home institutions and countries.

The discussions between the fellows and their mentors, done as part of the evaluation of the programme at the end of the fellowships, provided valuable insights into real-world challenges and learning outcomes, as outlined in Table 1. These in-depth focus group discussions, both face-to-face and online, shaped the authors’ collective viewpoint on the role of mentorship in advancing AMU surveillance in LMICs, which is central to this article.

**Table 1.** Summary of learning activities, outcomes and identified challenges

Learning activity	Outcomes	Identified challenges
Continuous interaction and engagement with mentors through regular (fortnightly) meetings over 12–24 months	Regular interactions with mentors enabled the fellows to stay on track in implementing their learning plan activities Strengthening mentor–fellow relationships fostered trust and created a supportive environment that promoted learning and growth Direct and active guidance on the job allowed the fellows to develop skills and address challenges in real time with personalized support	Inconsistent internet connectivity often disrupted online meetings Challenges in balancing fellowship with full-time work responsibilities Complications adapting learning plans iteratively based on changing needs, constrained by the fellowship monitoring framework
Literature review: country-specific antimicrobial surveillance	Developed problem statements for country Identifying common barriers and enablers Improvement in literature review and scientific writing skills	Gaps in evidence Limited ongoing surveillance Absence of local policies and guidelines
Apply skills to data analytic cycle, including: Data collection: gather antimicrobial data from systems along the pharmaceutical value chain Data cleaning and processing: transform the data into standardized format Data analysis: at national, provincial and hospital levels to identify patterns, relationships and insights Data visualization: communicate complex data insights using charts and graphs Data interpretation and communication: disseminate findings to diverse audiences	Develop surveillance strategies and implement protocols Data sources and their accuracy, relevance, and reliability Internationally accepted methodology—ensuring comparability of data across different countries Analysis of trends at hospital, provincial and country levels to provide useful insights for stakeholders Effective data presentation and visualization techniques to enhance understanding of the findings	Absence of electronic medical records, paper-based data collection a time-consuming task The lack of uniform data formats made data cleaning laborious Absence of data-sharing policies and mechanisms Lack of established data dissemination strategies hindered engagement with stakeholders, especially policymakers Lack of political/organizational leadership commitment gap between planning and implementation
Completion of training modules on AMS and treatment of common infections	Hybrid model blended online modules with in-person group discussions Gained foundational clinical skills and ability to conduct AMU audits	Modification to incorporate local issues into learning modules was needed
Training to conduct AMU audits using Australian tool known as the NAPS^15^	Conducted hospital audit to assess quality of AMU at a tertiary hospital	Difficulty in gaining approval and securing access to the hospital for audit process Lack of current treatment guidelines and trained infectious diseases physicians made it difficult to validate appropriateness assessments Paper-based medical records impeded audit efficiency Incomplete medical notes affected the accuracy of the audit
Participation in hospital visits in Melbourne, Australia to observe AMS programmes in action	Enabled knowledge transfer for implementing similar initiatives in their home countries	Organising hospital visits amid COVID-19 restriction (logistic obstacles, e.g. mask fit testing)
Attendance at AMS training workshops to help build workforce capacity within countries	Fellows transferred knowledge to others and contributed to broader capacity building in their home country	Competing national priorities and absence of directives on the need for antimicrobial surveillance
Collaboration with other fellows on common learning tasks	Shared learning tasks allowed insights from diverse perspectives, exposing the fellows to different policies and practices and broadening their knowledge base	
Attendance at international conferences	Allowed fellows to showcase their achievements, learn from experts and fostered an exchange of knowledge and experiences	
Participation in peer learning scientific writing discussions and writing retreats	Face-to-face writing workshops and online group meetings enabled the development of scientific writing skills	
Participation in meetings to explore antimicrobial surveillance system architecture across human and animal health	Served as a platform to develop a comprehensive understanding of one health while advancing interdisciplinary collaboration with animal health fellows	Meetings were sporadic

Based on their experience, the group highlighted several elements as being crucial to the success of a mentorship programme. The most important of these was the careful pairing of fellows (mentees) with mentors who possess the necessary expertise. Additionally, co-designing the mentee’s learning plan to align not only with institutional priorities and national AMR action plans but also with the comprehensive One Health Capacity Building and Training Framework.^15^ This framework was developed specifically for the fellowship programme by a team of mentors at the WHO CC for AMR and the Asia-Pacific Centre for Animal Health. It is structured around four key themes: technical expertise, leadership and organizational change, engagement and collaboration and translation and impact. Incorporating accountability mechanisms into these personalized learning plans featuring clear goals, tailored learning activities and realistic timelines helped ensure that both mentors and mentees adhered to their commitments and achieved the desired outcomes.

Moreover, we advocate for the development of the curriculum of fellowships, and the execution of teaching methods used within them, to adopt a multi-modal approach grounded in adult learning theory,^16^ recognizing that adult learners benefit most from engaging, directly applicable learning experiences tailored to their professional roles and work environments. This approach can encompass a range of methods, including online training modules that are moderated in real time by mentors, practical case studies that directly reflect mentee’s experiences, peer learning through group discussions, experiential learning during field visits, independent evaluation of evidence through self-directed study with reflection and discussion and collaborative projects such as writing retreats. For example, in our mentorship programme, we integrated online theoretical learning on AMS with in-person hospital-based training sessions focused on the utilization of AMU surveillance data for clinical and operational decision-making. These sessions were facilitated by mentors who provided guidance, feedback and opportunities for reflection. This blended approach allowed the mentees to receive comprehensive training, which was both theoretically rigorous and practically relevant to their roles.

In our view, a minimum duration of 12 months is needed for a mentorship programme in AMU surveillance. This period provides the intensity and depth required to comprehensively complete learning activities, including adequate training on identifying suitable data sources, collection methodologies, analysis, interpretation, visualization techniques and the timely implementation of dissemination strategies for surveillance findings. In addition, we learned that having fortnightly mentorship meetings was essential, as it provided a structure akin to having someone actively support learning in the workplace. These regular touchpoints fostered trust and continuous interaction, maintained momentum in completing tasks and promptly addressed challenges and evolving learning needs. They also created opportunities for iterative improvements and sustained engagement over the 12- to 24-month mentorship period.

To expedite training without the significant time investment required to develop survey tools and methodologies, it makes sense to leverage internationally established surveillance toolkits such as WHO GLASS or the National Antimicrobial Prescribing Survey (NAPS).^17^ Our mentorship programme supported the use of these tools, enabling the fellows to contribute to national AMU reporting within their respective countries as part of their roles as AMU surveillance focal points, as highlighted in the recent CAPTURA study.^18^ Furthermore, in today’s digital age, utilizing technology is essential for connecting learners from diverse locations, facilitating training and collaboration across vast distances. During the COVID-19 pandemic, technology, such as online meetings and webinars, sustained our mentorship programme by fostering continuous engagement and collaborative learning when international travel restrictions precluded in-person interaction.

We believe that recognition of their role as ‘Fleming Fund Fellows’ meant that the mentorship programme had credibility, encouraged home institutions to provide protected time to participate and motivated mentees to engage and successfully complete the programme. Formal recognition empowered the fellows to then become mentors within their own institutions, promoting knowledge transfer and ensuring sustainability of expertise. This approach builds local capacity while reducing reliance on international experts, often a more expensive alternative. Recently, several Fleming Fund Alumni Fellows have initiated local mentorship programmes in Nigeria, Ghana and Kenya.^19^ The advancement of technology is further driving innovative mentoring methods, including tele-mentoring models such as the ECHO programme’s TEACH-AMS initiative,^20^ which offer scalable solutions for LMICs.

Building on this, we contend with others that the benefits^21,22^ of mentorship should be recognized and embedded as a collective institutional responsibility,^13^ tailored to cultural contexts and progressively implemented in a phased science approach in LMICs.^11^ Over time, this strategy would enable the establishment of a local and regional network of mentors and mentees to sustain a thriving community of practice.

Finally, data from Cohort 1 of the Fleming Fund Fellowship Scheme across several countries showed that 100% of professional fellows in 2021 and 80% in 2022 made substantial progress in developing AMR-relevant competencies.^23^ We foresee that setting competency standards and providing guidance and mentorship toolkits^24^ adapted to local LMIC contexts will be necessary, and their implementation should be evaluated to ensure that scarce resources are applied to the most effective mentorship strategies.
